# Investigating The Relationships Between Subjective Cognitive Decline and Sleep Quality and Their Influence on Cognitive Functions

**DOI:** 10.1002/alz70856_106402

**Published:** 2026-01-08

**Authors:** Dwaiti Roy, Monisha S, Thomas Gregor Issac

**Affiliations:** ^1^ Centre for Brain Research, Indian Institute of Science, Bengaluru, Karnataka, India; ^2^ Centre for Brain Research, Indian Institute of Science, Bangalore, Karnataka, India

## Abstract

**Background:**

Subjective Cognitive Decline (SCD) is considered to be the crucial predementia stage and serves as one of the early indicators of dementia. It refers to the self‐perceived decline in cognition without any evidence of objective decline. On the other hand, studies have found that poor sleep quality has a higher chance of developing dementia in the later stages. Thus, in the present study, we aim to look at the relationship between SCD and sleep and their effect on cognition.

**Method:**

The present analysis was done using the baseline data of the Tata Longitudinal Study of Aging (TLSA) cohort. SCD was screened using a positive response to the memory question of the Geriatric Depression Scale (GDS) and scoring zero on the Clinical Dementia Rating (CDR) scale. An equivalent number of age, gender, and education‐matched healthy controls were selected for the study. The quality of sleep was assessed using the Pittsburgh Sleep Quality Index (PSQI) scale. Cognition was measured by the Hindi Mental Status Examination (HMSE) and Addenbrooke's Cognitive Examination (ACE‐III). Chi‐square test was done to check the association between SCD and sleep. The Generalized Linear Model (GLM) was used to assess the relationship between SCD and sleep with cognitive parameters.

**Result:**

The study utilized 140 participants with a mean age of 58.37±9.71 years. Among them, 70 participants were screened as having SCD. Chi‐square test revealed that SCD participants have a higher proportion of poor sleep quality when compared to their healthy counterparts (68.8% vs 31.3%, *p* = 0.001). Unadjusted GLM revealed that the group with poor sleep and SCD scored lower in HMSE (β=‐0.414, *p* = 0.025). When adjusted for Generalized Anxiety Disorder scale and GDS scores, the group with poor sleep and SCD scored less in the ACE attention task (β=‐0.951, *p* = 0.018)

**Conclusion:**

The findings suggest that poor sleep quality can impact cognitive functioning in the early stage of SCD. Therefore, implementing strategies for improving sleep quality can aid in delaying the onset of objective cognitive impairment.

